# Role of Essential Oil of *Mentha Spicata* (Spearmint) in Addressing Reverse Hormonal and Folliculogenesis Disturbances in a Polycystic Ovarian Syndrome in a Rat Model

**DOI:** 10.15171/apb.2017.078

**Published:** 2017-12-31

**Authors:** Mahmood Sadeghi Ataabadi, Sanaz Alaee, Mohammad Jafar Bagheri, Soghra Bahmanpoor

**Affiliations:** ^1^Department of Reproductive Biology, School of Advanced Medical Sciences and Technologies, Shiraz University of Medical Sciences, Shiraz, Iran.; ^2^Department of Anatomy, School of Medicine, Shiraz University of Medical Sciences, Shiraz, Iran.

**Keywords:** PCOS, Mentha spicata, Folliculogenesis, Rat

## Abstract

***Purpose:*** Given the antiandrogenic effects of spearmint, in this study we evaluated the effects of its essential oil on polycystic ovarian syndrome in a rat model.

***Methods:*** Female rats were treated as follows: Control, normal rats which received 150 mg/kg spearmint oil or 300 mg/kg spearmint oil, or sesame oil; and PCOS-induced rats which received 150 mg/kg spearmint oil or 300 mg/kg spearmint oil, or sesame oil. Then the animals were killed and the levels of LH, FSH, testosterone and ovarian folliculogenesis were evaluated.

***Results:*** Spearmint oil reduced body weight, testosterone level, ovarian cysts and atretic follicles and increased Graafian follicles in PCOS rats.

***Conclusion:*** Spearmint has treatment potential on PCOS through inhibition of testosterone and restoration of follicular development in ovarian tissue.

## Introduction

Polycystic ovary syndrome (PCOS) is an endocrine disorder associated with hyperandrogenism and elevated level of oxidative stress and, often, obesity, abnormal menstrual cycle, insulin resistance and oligomenorrhea or anovulation.^1^

An impressive number of plant species is traditionally used for treatment of fertility-related diseases.^2^ Currently in Iran, *Mentha spicata* (spearmint) essential oil is produced commercially and used orally as a carminative and antispasmodic agent. This herbal plant is also recommended for alleviating hirsutism and menstrual pain.

It is confirmed that spearmint tea has antiandrogen properties and significantly decreases testosterone level and hirsutism in women with PCOS.^3,4^ It also has antioxidant, anticancer, anti-inflammatory and antidiabetic properties,^5^ but the effects of spearmint oil on PCOS have not been determined. In the current study, we evaluated the effects of spearmint oil on an animal model of PCOS.

## Materials and Methods

### Preparation of Mentha spicata essential oil 

Purified *Mentha spicata* essential oil was purchased from Barij Essence Pharmaceutical Company, Mashhad Ardehal, Kashan, Iran and according to the manufacturer’s data sheet contained 57.02% carvone, 24.63% limonene, 2.7% pulegone, 1.8% menthol and 0.34% cineole.

### Animals

Mature Wistar albino female rats were obtained from the animal house of Shiraz University of Medical Sciences, Shiraz, Iran. Rats were kept in temperature-controlled rooms with constant humidity and 12 hr/12 hr light/dark cycle, with free access to standard diet and water. The study protocol was approved by the Animal Ethical Committee of Shiraz University of Medical Sciences.

Rats with two normal estrus cycles were weighed and treated as follows: Group I (control): Received 1 ml distilled water orally for 20 days; Group II: Received letrozole; Group III: Received letrozole and then received spearmint oil (150 mg/kg); Group IV: Received letrozole and then received spearmint oil (300 mg/kg); Group V: Received letrozole and then received sesame oil; Group VI: Received spearmint oil (150 mg/kg); Group VII: Received spearmint oil (300 mg/kg); and Group VIII: Received sesame oil. PCOS induction was carried out by treating rats in groups II, III, IV and V daily with letrozole orally (1 mg/kg) for 28 days and confirmed by observation of persistent estrus phase and a high number of ovarian cysts in ovarian histological sections. Spearmint oil was dissolved in sesame oil and administered to rats orally for 20 days.

After treatment duration, animals were weighed, killed by ether, blood sample was taken from the heart and the ovarian tissues were removed.

### Hormonal assay

Blood samples were centrifuged at 3000 rpm and Serum concentrations of testosterone (Padtan Elm Company, Tehran, Iran), LH and FSH (Hangzhao Eastbiopharm Co., Ltd., Hangzhao, China) were measured with their specific kits.

### Histological analysis

Ovaries were removed, fixed and sections of 5μm thickness were cut and stained with hematoxylin and eosin.‏ The number of primordial, primary, secondary, Graafian and atretic follicles, corpus lutea and cysts were counted in ovarian sections using a light microscope (Olympus, Tokyo, Japan).

### Statistical analysis 

Statistical analysis was performed using SPSS 16 software (IBM, Armonk, USA). For analysis of data, the One-Way ANOVA test was used followed by the Tukey test to compare the means. P value of <0.05 was considered statistically significant.

## Results and Discussion

The body weight, level of LH and FSH, testosterone and the number of primordial, primary, secondary, Graafian and atretic follicles and corpus lutea was not different among control and rats which received spearmint oil or sesame oil (p>0.05).

The weight of animals in the PCOS-induced group and in the PCOS-induced groups that received spearmint oil (150 mg/kg) and sesame oil was significantly higher than that of the control group (p=0.006, p=0.004 and p<0.001, respectively), but in PCOS-induced rats that had received the high dose of spearmint oil (300 mg/kg) the weight was significantly lower in comparison both to PCOS-induced rats (p=0.005) and to PCOS-induced groups that received spearmint oil (150 mg/kg) and sesame oil (p=0.003 and p< 0.001)(Table 1). Therefore, spearmint has no effects on body weight in normal condition, which was observed by other studies using spearmint extract,^6^ but in PCOS condition spearmint serves to control body weight.

**Table 1:** Body weight and the level of LH, FSH and testosterone of control, PCOS and PCOS rats which received spearmint oil

**Groups**	**Weight at the beginning (g)**	**Weight at the end (g)**	**LH (ng/dl)**	**FSH (ng/dl)**	**Testosterone (mIU/ml)**
**(I) Control**	161.33 ± 7.50	203.50 ± 14.05*	22.26±1.76	16.96±3.76	0.14 ± 0.02
**(II) PCOS**	149.00 ± 8.12	230.00±17.81	24.56±1.98	13.62±0.65	3.04 ± 0.69 †
**(III) PCOS+ Spearmint (150mg/kg)**	151.85 ± 6.46	235.57±12.36	21.88±4.13	13.61±1.64	0.20 ± 0.11
**(IV) PCOS+ Spearmint (300mg/kg)**	153.50 ± 7.23	205.00±10.70**	21.45±2.63	15.40±3.86	0.12 ± 0.01
**(V) PCOS +Sesame oil**	157.83 ± 12.87	244.16±10.90	25.85±3.49	14.50±0.88	0.14 ± 0.02

Data are shown as mean±SD. *P*<0.05 is considered statistically significant. * Significant differences between control and group II, group III and group V ** Significant differences between group IV and group II, III and V † Significant differences between group II and other groups

The level of testosterone in PCOS-induced groups that received spearmint oil was significantly lower than that of the PCOS-induced group (p<0.001) (Table 1). Studies have shown attenuation of testosterone in PCOS women after receiving spearmint teas.^3,4^

The number of primordial follicles in PCOS-induced rats and also in PCOS-induced rats that received either of two doses of spearmint oil and sesame oil was significantly lower than in the control group (p=0.016, p=0.046, p=0.001 and p=0.002, respectively). There was no significant change in the number of primary follicles among these groups (p>0.05).

The number of secondary follicles was significantly lower in the PCOS-induced group compared with the control group (p=0.043).

There were no Graafian follicles in the PCOS-induced group and in PCOS-induced groups that received the lower dose of spearmint (150 mg/kg) or sesame oil. However, in the PCOS-induced group that received the higher dose of spearmint, Graafian follicles were observed (p>0.05). The number of atretic follicles was significantly higher in the PCOS-induced group compared with the control group (p< 0.001), but in the two PCOS-induced groups that received spearmint oil, the number of atretic follicles decreased significantly in comparison to the PCOS-induced group (p=0.008 and p=0.011). The number of corpus lutea was significantly lower in the PCOS-induced group compared with control group (p=0.003), but it was insignificantly higher in the PCOS-induced groups that received spearmint oil in comparison to the PCOS-induced group (p>0.05). In PCOS-induced groups that received spearmint oil‏ or sesame oil, the number of ovarian cysts was significantly lower than in the PCOS-induced group (p< 0.001). In the PCOS-induced group which received high doses of spearmint oil, no cysts were observed (Table 2).

It is demonstrated that in PCOS condition, obesity, insulin resistance and hyperglycemia all correlate with a high level of oxidative stress, inducing a hyperandrogenemic environment in the ovary. Although locally produced androgens serve as substrate for estrogen production in folliculogenesis, an excessive level of androgens overrides follicular development, resulting in follicular atresia, disturbed follicular development and anovulation.^7^

Elevated visceral adiposity and hyperinsulinemia are observed in PCOS women, resulting in increased androgen production of the ovaries and adrenal gland. Reducing body weight of anovulatory obese women decreases insulin resistance, testosterone concentration and restores ovulation.^8^

**Table 2:** The number of primordial, primary, secondary, Graafian and atretic follicles, corpus lutea and cysts in ovarian tissue of control, PCOS and PCOS rats which received spearmint oil

**Groups**	**Primordial follicle**	**Primary follicle**	**Secondary follicle**	**Graafian follicle**	**Atretic Follicles**	**Corpus Luteum**	**Cysts**
**(I) Control**	8.41 ± 5.48 *	8.72 ± 7.76	6.33± 2.57	0.16 ± 0.38	2.66 ± 2.26	7.25 ± 3.64	0.00
**(II) PCOS**	2.83 ± 2.99	2.66 ± 2.16	1.33 ± 1.21**	0.00	15.83±9.96†	1.33±1.03**	10.50±4.50§
**(III) PCOS+Spearmint(150 mg/kg)**	3.20 ± 3.76	4.20 ± 3.70	2.20 ± 3.03	0.00	2.60 ± 2.30	4.80 ± 3.56	0.20 ± 0.44
**(IV) PCOS+Spearmint(300 mg/kg)**	2.14 ± 2.11	5.28 ± 2.98	2.85 ± 2.11	0.14 ± 0.03	4.00 ± 4.43	4.28 ± 2.42	0.00
**(V) PCOS+Sesame oil**	2.000 ± 1.67	2.500±2.05	2.00 ± 1.20	0.00	10.16 ± 8.49	2.83 ± 2.22	1.66 ± 2.25

Data are shown as mean±SD. *P*<0.05 is considered statistically significant. * Statistically significant differences between control and other groups ** Significant differences between group II and control groups † Significant differences between group II and control, group III and group IV § Significant differences between group II and other groups

According to our results, spearmint oil decreases body weight in the PCOS condition, and since it has antiandrogenic potential, its administration leads to decrease of androgen production. Studies show that spearmint leaves decrease cholesteroland, in type II diabetes, decrease oxidative stress.^9^ Additionally, phenolic compounds of spearmint leaf extract significantly enhance the antioxidant defense system and reduce body weight and levels of glucose and cholesterol in diabetic male rats.^10,11^

In the current study, no Graafian follicle was observed in PCOS-induced rats and only the administration of the higher dose of spearmint resulted in the production of these follicles in the ovarian tissue. Furthermore, administration of spearmint decreased the number of atretic follicles and ovarian cysts in PCOS-induced rats, a circumstance which is also associated with the antioxidant and antiandrogenic effects of spearmint oil. Moreover, the attenuated level of corpus lutea in PCOS-induced rats increased in PCOS rats that received spearmint oil, which reflects the higher rate of ovulation in these groups.

Therefore, spearmint oil by reduction of weight and testosterone and having antioxidant potential can restore follicular maturation and induce ovulation, which, respectively, was observed in the lower number of atretic follicles and higher number of Graafian follicles and corpus lutea in the PCOS-induced rats that received spearmint oil.

## Conclusion

Spearmint can be administered as a potential agent for treatment of PCOS, but further research is needed to examine the effects of this herbal plant on all parameters related to fertility.

## Acknowledgments

We thank Shiraz University of Medical Sciences for the ethical approval and financial support of this research (grant number:93-01-74-8667). 

## Ethical Issues

The study protocol was approved by the Animal Ethical Committee of Shiraz University of Medical Sciences

## Conflict of Interest

The authors declare that there are no conflicts of interest.
